# Circuitry and Synaptic Dysfunction in Alzheimer's Disease: A New Tau Hypothesis

**DOI:** 10.1155/2020/2960343

**Published:** 2020-09-01

**Authors:** Siddhartha Mondragón-Rodríguez, Humberto Salgado-Burgos, Fernando Peña-Ortega

**Affiliations:** ^1^CONACYT National Council for Science and Technology, México, Mexico; ^2^UNAM Developmental Neurobiology and Neurophysiology, Institute of Neurobiology, National Autonomous University of México, Querétaro, Mexico; ^3^UADY Neurosciences Department, Autonomous University of Yucatán, 97000 Mérida, Yucatán, Mexico

## Abstract

For more than five decades, the field of Alzheimer's disease (AD) has focused on two main hypotheses positing amyloid-beta (A*β*) and Tau phosphorylation (pTau) as key pathogenic mediators. In line with these canonical hypotheses, several groups around the world have shown that the synaptotoxicity in AD depends mainly on the increase in pTau levels. Confronting this leading hypothesis, a few years ago, we reported that the increase in phosphorylation levels of dendritic Tau, at its microtubule domain (MD), acts as a neuroprotective mechanism that prevents N-methyl-D-aspartate receptor (NMDAr) overexcitation, which allowed us to propose that Tau protein phosphorylated near MD sites is involved in neuroprotection, rather than in neurodegeneration. Further supporting this alternative role of pTau, we have recently shown that early increases in pTau close to MD sites prevent hippocampal circuit overexcitation in a transgenic AD mouse model. Here, we will synthesize this new evidence that confronts the leading Tau-based AD hypothesis and discuss the role of pTau modulating neural circuits and network connectivity. Additionally, we will briefly address the role of brain circuit alterations as a potential biomarker for detecting the prodromal AD stage.

## 1. Introduction

Alzheimer's disease (AD) is a public health problem for our aging societies and is histopathologically defined by extracellular amyloid-beta (A*β*) deposits and intracellular hyperphosphorylated Tau (pTau) deposits [1–7]. Although many areas of the brain are affected during AD development, the hippocampus, a circuit related to spatial orientation and cognitive functions, is a major focus of AD research attention [1, 2, 5–7]. Current AD hypotheses postulate that (a) due to A*β* increase, Tau becomes abnormally phosphorylated, and (b) pTau dissociates from microtubules and (c) aggregates into neurofibrillary tangles (NFTs) [3], causing neuronal dysfunction and, eventually, cell death [4]. In support of its pathological role, pTau has been directly linked to several neurodegenerative disorders (tauopathies), such as AD, frontotemporal dementia, Parkinson's disease, Down syndrome, and Pick's disease [5–7].

As mentioned, abnormally phosphorylated Tau is a key mediator of A*β*-induced dendrite dysregulation and synaptic dysfunction [8, 9]. In this regard, a study performed with AD patients who had relatively low levels of A*β* and high levels of Tau showed dysfunction in synaptic plasticity and a faster cognitive decline [10]. Thus, considering that pTau aggregates correlate better with cognitive impairment in AD than A*β* aggregates [11], pTau has emerged as the new therapeutic target against AD and related neurodegenerative disorders [12]. Intensive research focusing on the pathological role of pTau has strongly nurtured the use of pTau as a therapeutic target [12, 13]. These therapeutic approaches have been restricted to a reduction of Tau levels and lowering activity of kinases that phosphorylate Tau [12, 13]. However, as for the numerous AD therapies that have aimed towards A*β*, we predict that the Tau-based strategy will render mild outcomes. To justify this hypothesis, it is critically important to discuss not only the pathological but also the physiological functions of pTau [13, 14], namely, its recently described synaptic functions [13–15]. By doing so, we will confront and update the current Tau-based AD hypotheses. Additionally, this review will highlight the novel pTau function of regulating neural activity and preventing Aß-induced synaptic overexcitation [13, 14, 16]. This review also appraises the available evidence on neural network dysfunction in preclinical and confirmed AD, identifies research gaps on current AD hypotheses, and points towards new research directions to understand and treat this disease.

## 2. Tau Phosphorylation and Synaptic Plasticity

**Figure 1:**
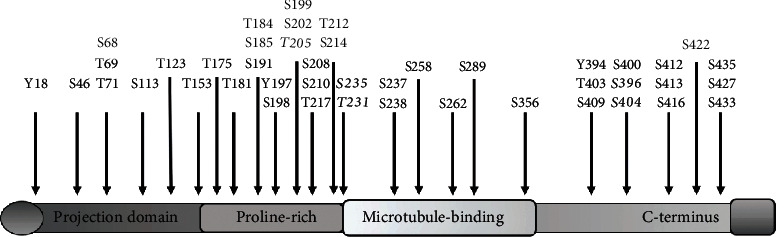
Tau sequence can be subdivided into two active domains that are phosphorylated throughout. Tau protein contains an assembly domain (carboxy-terminus section that contains the microtubule-binding and flanking regions; right). It also contains a middle region (comprising the proline-rich domain that contains multiple Thr-Pro of Ser-Pro motifs). Finally, Tau contains the amino-terminal section (left).

**Figure 2:**
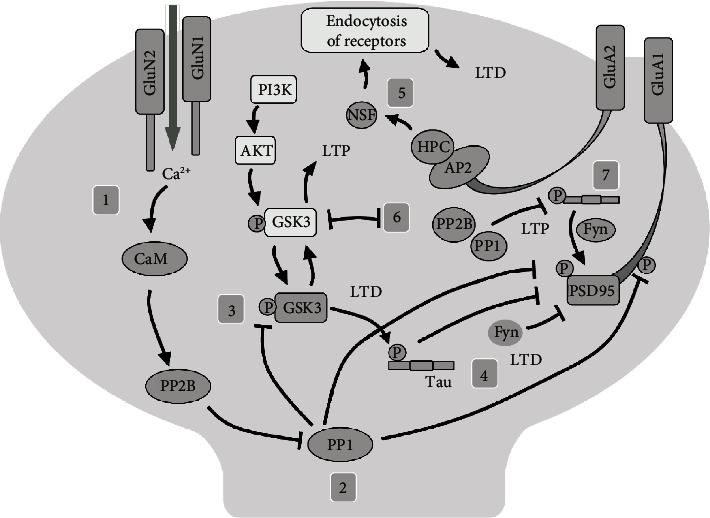
Tau phosphorylation and its involvement in long-term depression (LTD). (1) Calmodulin (CaM) detects a Ca^2+^ increment due to NMDAr activation, leading to protein phosphatase-2B (PP2B) activation and the subsequent activation of the PP1. (2) PP1 dephosphorylates Ser845 at the AMPAr subunit GluA1, Ser295 at PSD95, and GSK3*β*. (3) Active GSK3*β* leads to pTau near MD (microtubule domain). (4) pTau induced LTD by the dissociation of Tau/Fyn/PSD95 complex. (5) Dissociation of Tau/Fyn/PSD95 leads to disruption of interaction between GluA2 and with N-ethylmaleimide-sensitive factor (NSF) that cause clathrin-mediated endocytosis of receptors during NMDAr-LTD. (6) Inactive GSK3*β* (through the PI3K/AKT pathway) and dephosphorylated Tau (that recruits Fyn kinase to target the PSD95/NMDAr complex) promote long-term potentiation (LTP) (arrow = activation, flat line = inhibition).

Tau is a microtubule-associated protein that promotes microtubule stability and function [17]. Tau is encoded by the microtubule-associated Tau gene, which comprises 16 exons on chromosome 17q21 [17]. Tau has more than 45 phosphorylation sites located in its proline-rich domain (Figure 1, residues 172-251) and C-terminal domain (Figure 1, residues 368-441) [2, 5, 6, 13–15]. Additionally, Tau has a microtubule domain (MD) segment that functions as a protein-protein binding domain (Figure 1, microtubule binding), therefore promoting microtubule stabilization and regulating the structure and proper function of neurons [17]. It is well documented that Tau protein is principally located in the soma and axons of neurons [3, 17]. However, recent evidence showed that Tau is also a dendritic protein [13–15]. Specifically, it was confirmed that endogenous Tau localizes at the postsynapses in neurons under physiological conditions [14]. This raised an important question: what is the role of Tau protein at the postsynapsis? According to recent data, it appears that dendritic Tau modulates plastic mechanisms involved in memory storage [8, 9, 14–16]. At the physiological and molecular levels, long-lasting synaptic plasticity changes are considered the cellular correlates of memory storage [18–22]. Among those synaptic plasticity phenomena related to memory, synaptic strength can be long-lasting enhanced (long-term potentiation (LTP)) or long-lasting depressed (long-term depression (LTD)), and these changes can persist from hours to days [18–22]. The cellular mechanisms underlying LTD, depicted in Figure 2, are triggered by N-methyl-D-aspartate receptor (NMDAr) activation at the synapse [18–22]. Broadly, this process involves the following steps: (1) calmodulin (CaM) binds Ca^2+^, increased via NMDAr, which leads to CaM/Ca^2+^-dependent protein phosphatase 2B (PP2B) activation [21], which in turn leads to protein phosphatase 1 (PP1) activation through the dephosphorylation of its inhibitor-1 [21]. Then, (2) activated PP1 dephosphorylates key targets required for LTD, such as Ser845 at the AMPAr subunit GluA1 [21, 22], Ser295 at the postsynaptic density protein 95 (PSD95), and activates glycogen synthase kinase-3*β* (GSK3*β*) [23, 24] (Figure 2). (3) Activated GSK3*β* phosphorylates Tau protein at flanking MD [25] (Figures 1 and 2), (4) leading to the dissociation of Tau/Fyn (a member of the Src-tyrosine kinase family)/PSD95 complex [13, 14]. Interestingly, we have observed that changes in the Tau phosphorylation levels, in sites near the Tau's MD, modulate its interaction with PSD95 and Fyn kinase [13, 14] (Figures 1 and 2). Specifically, we found that increasing pTau at sites such as Ser396, Ser404, Thr205, Thr231, and Ser235 (Figure 1 and 2) promoted dissociation of the Tau/Fyn/PSD95 complex, which is a determinant for LTD induction [13, 14]. In the same regard, it was reported that Tau phosphorylated at Ser396, followed by activation of NMDAr, is necessary for the expression of hippocampal LTD (i.e., pTau shifts synaptic plasticity from LTP to LTD) [15]. Altogether, we proposed that phosphorylation of Tau's MD is a crucial part of a regulatory mechanism that controls NMDAr activity [13, 14]. In other words, non-phosphorylated Tau contributes to LTP, while pTau contributes to LTD [9, 13–16]. Additionally, (5) dissociation of the Tau/Fyn/PSD95 complex promotes the loss of its interactions with GluA2 and with N-ethylmaleimide-sensitive factor (NSF) that cause clathrin-mediated endocytosis of receptors during NMDAr-LTD [18, 22, 26]. (6) In opposition, phosphoinositide 3-kinase (PI3K), in an AKT-dependent pathway, can modulate GSK3*β* activity [27], allowing PP1 and PP2B to dephosphorylate Tau protein at the following sites: Ser199, Ser202, Thr205, Thr212, Ser214, Ser235, Ser262, Ser396, Ser404, and Ser409 [28]. (7) Dephosphorylated Tau recruits Fyn to target the PSD-95/NMDAr complex, leading to LTP induction [13, 14, 22, 29] (Figure 2).

Summarizing, we proposed that phosphorylation of Tau's MD is a crucial part of a physiological regulatory mechanism that controls NMDAr activity and synaptic coupling [13, 14, 29].

## 3. Amyloid-Beta as an Effector of Phosphorylated Tau

The proteolysis of amyloid precursor protein (APP) into aggregation-prone A*β* peptides (mainly of 40 or 42 amino acids) has long been implicated in the etiopathogenesis of AD [1, 8–14, 30–36]. In the vast body of evidences that supports the synaptotoxic role of A*β*42 oligomers and its consequences, there are key observations: A*β*42 oligomers extracted from the late-onset AD brains (1) inhibit LTP [30], (2) enhance LTD [13, 14, 29, 31], (3) impair memory [30], (4) decrease synapse density [32], (5) reduce the number of NMDAr on the cell surface at synapses [33], and (6) interfere with the reuptake of extracellular glutamate [34]. Additionally, (7) A*β*42 oligomers induce Tau hyperphosphorylation at AD-relevant sites (near Tau's MD) [14, 35] that (8) cause excitotoxicity and neuritic dystrophy [35, 36]. In our hands, the application of monomeric A*β*42 to rat slice cultures leads to the formation of A*β*42 oligomers over the incubation time of 5 days [14], which correlates with increased pTau at Ser396, Ser404, Thr231, and Ser235 sites [14]. Our data showed that A*β*42 oligomers initially induce the phosphorylation of a few specific sites on Tau, rather than increase global Tau phosphorylation at all sites, as seen in advanced AD [37]. Importantly, the early phosphorylated sites are close to the Tau's MD (Figure 1). This is consistent with other reports where phosphorylation of Tau protein at Ser396/Ser404 sites was found as one of the earliest phosphorylation events during AD and related tauopathies [2, 5–7]. Further supporting the above findings, significant increases of Tau protein at the Thr231 site was reported in human patients during the early stages of AD development [38]. Indeed, it was reported that abnormal Tau processing is characterized by a sequential phosphorylation as follows: (1) Thr231, (2) Ser 202/Thr205, and (3) Thr212/Ser214 [38]. Of relevance, it is well established that Tau phosphorylation promotes the formation of tangle-like filament morphology [39, 40] and proaggregant Tau actively contributes to impaired memory and loss of LTP [8, 9, 41]. These results explain the weakening of synapses that morphologically would result in a loss of dendritic spines and functionally in a loss of memory [41–43]. Thus, indicating that the ability of Tau to aggregate is a crucial factor in disease development [2–6, 39–44]. However, a recent study showed that intracerebroventricular injection of Tau soluble aggregates, but not monomers or fibrils, increased the threshold for LTD induction in a prion protein-dependent manner [45]. Interestingly, Tau soluble aggregates blocked A*β*-induced LTD facilitation, whereas a subthreshold dose of Tau soluble aggregates promotes A*β*-induced LTP inhibition [45]. The data suggest that Tau soluble aggregates reduce the dynamic range of synaptic plasticity and that the prion protein acts as a common mediator of the synaptotoxicity induced by soluble A*β* and Tau [45].

Further supporting the mechanistic link between A*β* and Tau, injection of synthetic A*β* into the brain of transgenic mice that accumulate NFTs, owing to the overexpression of human Tau with a P301L mutation, induced a five-fold increase in the number of NFTs in regions near the injection sites [44]. Although the molecular mechanism functionally linking A*β*42 to Tau, as well as its contribution to the pathophysiological mechanism behind AD progression, remains under extensive study, it was reported that A*β*42 increased pTau and that GSK3*β* inhibition blocked the increased pTau and prevented A*β*42-induced impairment of LTP in mice [9]. It is known that GSK3*β* directly phosphorylates Tau on Thr181, Ser202, Thr205, Thr231, Ser396, Ser400, and Ser404 [25, 40, 46]. Thus, there is evidence for increased activation of GSK3*β* in human patients during early stages of AD [3, 37, 47, 48]. Based on that, deregulation of GSK3*β* has been proposed as a center-stage event linking extracellular A*β* and intracellular Tau protein [3, 48]. In this regard, it was reported that transgenic mice expressing a phosphorylation defective mutant GSK3*β* show impaired memory, impaired hippocampal LTP, and facilitated LTD [48]. Mechanistically, it has been proposed that during AD and related dementias, GSK3*β* activity may become deregulated due to its increased expression or as a result of alterations in upstream regulators of GSK3*β*, leading to enhancement of NMDAr-LTD and neurodegeneration [18].

Although it is not entirely clear how A*β*42 initiates the toxic cascade that causes all the synaptic alterations and the accumulation of proaggregant pTau, the activation of GSK3*β* seems to be the crucial executor of A*β*42 toxicity.

## 4. Tau and Synaptic Dysfunction

A recent study demonstrated that A*β*42 oligomers induce *de novo* synthesis of Tau protein, and its hyperphosphorylation at multiple residues, in the somatodendritic compartment, which is mediated by the Fyn/ERK/S6 signaling pathway [49]. This observation further supports an alternative mechanism for AD synaptic dysfunction, mediated by the aberrant translation and cellular redistribution of Tau protein in the somatodendritic region, which is due to the overstimulation of AMPA and NMDA receptors, as reported [50]. Indeed, the presence of Tau-mRNA in a dendritic ribonucleoprotein (RNP) complex allows for its local translation upon glutamatergic stimulation [50]. The interaction of Tau-mRNA containing RNP with Myosin Va, a postsynaptic motor protein, suggests that Tau-mRNA is transported into dendritic spines [50].

In addition to the alternative mechanism just described, a widely accepted theory for AD synaptic dysfunction proposes that A*β*42 oligomers induce Tau translocation from the axon to the dendritic spines causing early synaptotoxic effects (excitotoxicity) and progressive dendritic spine loss [8, 51, 52]. Supporting this theory, it was reported that oligomeric A*β*42 increases the redistribution of Tau to the somatodendritic compartment in AD mouse models [51, 52]. Thus, overexpression of Tau in cultured neurons and AD mice increases Tau in the somatodendritic compartment [51]. Importantly, the increased postsynaptic Tau was linked to spine loss in a Tau transgenic mouse model [51, 52]. Additional results showed that A*β*42 oligomers, through NMDAr, increased intracellular Ca^2+^ and triggered activation of AMP-activated kinase (AMPK), which leads to increased pTau (sites Ser262, Ser356, and Thr231) and dendritic spine loss [52–54]. In related studies, soluble A*β* oligomers, at picomolar concentrations, disrupt hippocampal LTP in slices and *in vivo* and impair the memory of complex learned behaviors in rodents [55]. A*β* oligomers also decrease dendritic spine density in organotypic slice cultures and contribute to excitotoxicity [55].

It is well known that a major component of the excitotoxicity process is the overactivation of NMDAr [56, 57]. NMDArs are heteromeric complexes formed by subunits NR1, NR2A, and NR2B [18, 56, 57]. Fyn kinase phosphorylates the NR2B subunit, which facilitates its interaction with PSD95 [58, 59]. It has been proposed that this interaction increases NMDAr stability and activity [18, 58, 59]. Furthermore, excess Fyn upregulates NMDAr activity, therefore affecting the levels of Ca^2+^ and causing calcium-driven excitotoxicity [8, 16, 52]. Additionally, it has been reported that there is a disturbance in intracellular Ca^2+^ homeostasis via changes in the calbindin D_28K_ protein, causing a reduction in the number of cells and a shrinking of its dendritic trees [60]. Furthermore, it has been reported that increased Fyn correctly targeted to the postsynaptic compartment causes memory deficits, excitotoxicity, and seizures and induces premature mortality in APP transgenic mice [8, 52]. Mechanistically, this can be explained by Tau protein directly binding to Fyn kinase; therefore, a Tau-dependent increase of Fyn, in dendritic spines, enhances excitotoxic signaling [8, 52].

Acute excitotoxicity is not the only Tau-dependent effect of A*β*42 oligomers. A*β*42 oligomers also have been found to cause Tau microtubule disassembly and ectopic cell-cycle reentry, which leads to neuronal death [61]. Because microtubules are essential for the efficient delivery of synaptic components to axon terminals and postsynaptic components to dendritic terminals [61], the A*β*-induced Tau-dependent effects on microtubules constitute important threats to synaptic function.

Based on these findings, several groups have proposed the elimination of Tau protein as a potential therapeutic approach that could aid in mitigating AD disease progression [8, 9, 12]. In line with this hypothesis, it was reported that the absence of Tau disrupts postsynaptic targeting of Fyn kinase, which correlates with reduced NMDAr-mediated excitotoxicity and hence mitigation of A*β*-induced toxicity [8]. Additionally, knocking out the endogenous Tau gene relieved the APP mice from memory deficits, seizures, and premature mortality [8]. In addition, it was reported that the absence of Tau protein prevented A*β*-induced impairment of LTP [9].

Although all these interactions remain under extensive study, the exact mechanism of coupling between these two proteins is still unknown. One proposed pathological interaction between A*β*42 and pTau comprises the following cascades: (1) A*β*42 causes abnormal activation of Tau kinases [3, 9, 14, 35, 40, 47, 52, 55], (2) activated Tau kinases cause abnormal phosphorylation of Tau protein at several sites [3, 14, 25, 35–37], and finally, (3) the abnormally phosphorylated Tau contributes to excitotoxicity [8, 62]. Therefore, scientists have proposed the blockade of Tau kinases as a potential therapeutic target against AD [9, 12, 13]. The rationale is simple: blocking Tau kinases will lead to non-phosphorylated Tau and, consequently, to less cytotoxicity [9, 12, 13].

## 5. Tau and Neuronal Network Activity

Cognitive capacities rely on a variety of brain network activity patterns, which represent distinct operating brain modes that are closely linked to fine changes in neuronal synchrony and are adjustable to behavioral demands [63, 64]. Therefore, alterations in synchronized network activities could help to explain cognitive deficits observed during AD development and tauopathies [65]. In this regard, recent studies support the early network dysfunction (neuronal hyperactivity, altered oscillatory activity, and loss of neuronal synchrony) as a crucial event that may lead to neurodegeneration and AD development [63–69]. In line with this hypothesis, it has been reported that hippocampal and cortex hyperactivity, which increases the risk of seizures, is presented in the early stages of AD [70, 71]. Indeed, convulsive seizures occur in over 80% of patients with an early stage of AD [71]. Thus, studies reported spontaneous epileptic activity with reduced gamma oscillations in AD [71, 72]. Notably, this activity depends on the inhibitory synaptic activity provided by the GABAergic parvalbumin-positive (PV+) interneurons [70, 73]. Increasing repetitive firing of PV+ cells favors gamma oscillations of neural networks [73–77]. In consequence, if the neural network synchronization is primarily controlled by the PV+ cells, then the disinhibition of principal cells (PCs) may produce less organized network activity leading to impaired information processing and cognitive function.

It has been hypothesized that the modifications in synaptic neuronal circuitries and the cognitive alterations in AD are produced by damage to PV+ interneurons that control the fine-tuning of neuronal network oscillations [76–81]. According to this hypothesis, the brain network alterations, rather than protein deposition, could account for the early pathogenesis of the disease development [67, 68]. Although the exact causes and mechanisms of network alterations have not been defined, interneuron dysfunction has emerged as a strong candidate [79–82]. Additional evidence suggests that GABAergic transmission of the PV+ cells is altered in AD, especially due to modifications in the function and number of cells; i.e., bodies and axons suffer size reduction, whereas the number of PV+ cells suffered the loss of over 70% in AD postmortem patients [82]. The cellular and molecular basis of this interneuron dysfunction has been studied in AD mouse models [67, 68, 70, 79]. For example, since gamma oscillations are mainly generated by PV+ cells, through their direct control of PCs firing [83], and since alterations in PV+ cell activity affect gamma oscillations and memory in AD mouse models [68, 69, 84], the rescue of PV+ cell activity promises the restoration of oscillatory activity, network synchrony, and memory [80, 84]. Notably, mouse models and humans with AD had reduced levels of voltage-gated sodium channels subunit Nav1.1 in interneurons [84]. Thus, restoring PV+ cell activity through the overexpression of these channels increased inhibitory activity and restored oscillatory activity in AD mouse models [80]. Additionally, the restoration of PV+ cells contributed to the reduction of hypersynchrony, memory deficits, and premature mortality of AD mouse models [80]. In our hands, we found that oscillatory activity was significantly less rhythmic in a young (post-natal day 30) AD mouse model [68]. Additionally, AD mice displayed significantly less coupling between slow (2-10 Hz) and fast (25-250 Hz) oscillatory frequencies. Mechanistically, we found that the intrinsic excitability of PV+ cells was significantly reduced in the p30 AD mouse model [68]. Because PV+ cells play a vital role in coordinating hippocampal synchrony between slow and fast oscillations [85], restoring PV+ cell activity could also help to restore the brain synchrony and cognitive functions in early stages of AD.

Following the same line of thought, recent data showed that enhancing G-protein-coupled inwardly rectifying potassium (GirK) channels, which would contribute to neuronal inhibition, can control the excess of neuronal excitability [86, 87]. Indeed, intracerebroventricular injection of A*β*42 or the GirK channel blocker tertiapin impaired GABAA-mediated field potentials and GirK-mediated field potentials, as well as its LTP [86], which is prevented by the application of the GirK channel activator ML297 [86]. The GirK channel activator ML297 also rescues glutamatergic LTP and habituation memory from its inhibition induced by A*β*42 [87].

So far, the described data have established the potential role of changes in inhibitory function affecting brain network activity during the early stages of AD development; however, the role of Tau protein in this process and in neural network dysfunction overall remains to be discussed. In this regard, it has been shown that abnormal Tau disrupts the ongoing network activity of neocortical pyramidal cells before cell death [88]. Interestingly, abnormal Tau decreased the activity of subpopulations of neurons, which, in turn, reduced the activity of the neocortical networks [89]. In line with these findings, aggregation of abnormally phosphorylated Tau protein in the entorhinal cortex (EC) was enough to disrupt the coordination of local field potentials between its efferent regions [89]. At the cellular level, it is known that the application of Tau soluble oligomers in the CA1 region of the hippocampus and layer VI of the neocortex increases the input resistance, reduces the amplitude of the action potential, and slows the rise and decay times of the action potential, without modifying synaptic transmission, but impairing the induction of LTP [90]. Furthermore, Tau oligomers isolated from human AD patients produced an inhibition of the hippocampal LTP and induced memory dysfunction in rodents [91]. In our hands, we found that PCs and PV+ cells in p30 triple-transgenic AD mice accumulate pTau at MD sites and that this accumulation correlated with changes in hippocampal oscillatory activity [92, 93]. Taken together, the latest evidence supports pTau accumulation in PCs and interneurons as an essential event that contributes to firing instability and, therefore, to brain network alterations, especially during very early stages of disease progression.

In summary, the A*β*42 oligomers and pTau induce changes in synaptic functions of PCs and interneurons and compromise the function of neuronal networks (i.e., long-term synaptic plasticity and synchronized networks).

## 6. Classical Tau Hypothesis versus New Hypothesis

**Figure 3:**
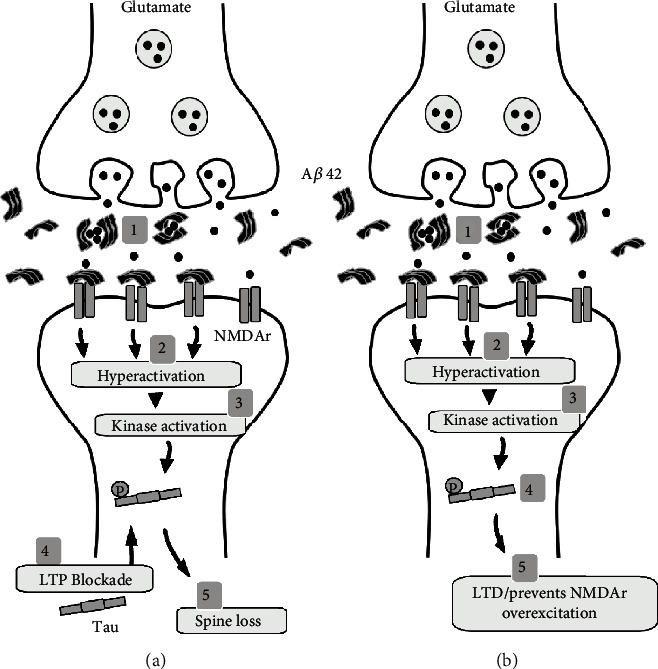
pTau protein at MD (microtubule domain) prevents NMDAr overexcitation, which accounts for protection rather than damage. (a) A*β*42 oligomers repeatedly affect neurons by blocking glutamate reuptake (1), which leads to hyperactivation (2), kinase activation (3), Tau hyperphosphorylation, and Tau translocation from the axon to the dendritic spines (4) causing LTP blockade and progressive spine loss (5). (b) Alternatively, A*β*42 oligomers block glutamate reuptake (1), which leads to hyperactivation (2), kinase activation (3), and Tau phosphorylation at MD sites (4), which alternatively promote LTD, preventing further NMDAr-mediated overexcitation and producing neuroprotection (5).

As previously discussed, a current model of AD synaptic dysfunction proposes that (1) A*β*42 oligomers induce abnormal kinase activation, (2) leading to Tau hyperphosphorylation and (3) translocation from the axon to the dendritic spines causing (4) LTP blockade, (5) progressive synaptic dysfunction, (6) progressive dendritic spine loss, and (7) early network dysfunction. Adding to the synaptic/network dysfunction, it was recently reported that A*β*42 oligomers block glutamate reuptake and overactivate NMDAr, leading to hyperactivation and neurodegeneration during early stages of disease progression [34, 94] (Figure 3(a)). Thus, from the classical point of view, all the data argued so far could favor the existence of a toxic cascade of A*β*42-pTau/synaptic damage/network dysfunction. However, we firmly believe that our data, along with recently published data, will help to unveil a new hypothesis in which Tau phosphorylated near the MD could have a protective role during the early stages of AD development [13–16, 29, 92, 93]. One molecular basis for this alternative hypothesis is that pTau protein at MD sites is physiologically located at the synaptic terminals [13, 14, 29]. Furthermore, pTau protein at MD sites is not exclusively translocated from axons to dendrites, as suggested by the classical hypothesis [3, 8, 49, 50, 52]. Second, pTau protein at MD sites prevents NMDAr overexcitation [13, 14, 29], which accounts for protection rather than damage, at the very least, during the early stages of disease progression. If our alternative hypothesis is true, it would be expected that the induction of epileptic activity, partially mediated by NMDAr hyperexcitation, could be prevented by elevating levels of pTau at MD sites. It has to be remembered, the prevalence rates of seizures are significantly increased in patients with AD [95–97]. In fact, we confirmed this by challenging p30 triple transgenic AD mice with the potassium channel blocker 4-aminopyridine, a potent proepileptic drug [92, 93]. We found that the p30 triple-transgenic AD mice exhibited a dramatic reduction in seizure-like activity after the 4-aminopyridine application [92, 93]. We also found that this reduction in the induction of epileptic activity correlated with pTau's MD accumulation in PCs [92]. Collectively, our results provide support for the alternative role of pTau protein during normal physiological conditions and early stages of AD development [7, 13, 14, 92, 93]. Importantly, it was reported that during the early stage of AD development, phosphorylation of Tau protein at its site Thr205 alleviated A*β*-induced neuronal death and provided protection from excitotoxicity [16]. Thus, an AD model with elevated pTau is more resistant to seizure activity induced by the proepileptic drug pentylenetetrazole [16]. Further supporting our hypothesis, and in agreement with our previously published data [14], this reduction in hyperexcitability is related to an increase in pTau-dependent disruption of PSD95/Tau/Fyn interactions and to the inhibition of A*β*-induced toxicity [16]. Altogether, these new pieces of evidence support a novel protective role of pTau (mainly by preventing NMDAr overexcitation) during early stages of AD development.

## 7. Conclusions and Perspectives

For the past decade, we have accumulated evidence that supports an alternative pTau hypothesis during the early stages of disease progression. The following is a summary of this evidence: (1) Tau phosphorylation at specific sites (near MD) appears during early stages of disease progression in the brain tissue from AD, Parkinson's disease, Down syndrome, and frontotemporal dementia cases [2, 5–7, 98, 99]. (2) These phosphorylation events precede early truncation events in Tau protein (i.e., truncation at site Asp421) and early conformational changes (i.e., folding from the amino terminus to the MD) [2, 5, 6, 98, 99]. (3) The phosphorylation events occur prior to the apparition of the classical fibrillar structures (NFTs and A*β* plaques) [2, 5, 6, 98, 99]. (4) Endogenous pTau near MD sites is physiologically located at postsynaptic sites where it interacts with the PSD95-NMDAr complex [13, 14, 29]. We showed that Tau binds directly to PSD95 protein [13, 14, 29]. (5) Increases in pTau at MD sites serve as a regulatory mechanism that prevent NMDAr overexcitation [13, 14, 29, 92]. (6) Increased pTau at MD sites confers protection from epileptic activity by reducing hippocampal excitability [13, 14, 92]. Finally, (7) we showed that increases in pTau at MD sites confer protection by disrupting PSD95/Tau/Fyn interaction and promoting LTD [13, 14, 29].

Collectively, our data, along with current published data, nurture the alternative pTau hypothesis [13, 14, 29, 92]. However, many scientific efforts are needed to further support the nonpathological role of pTau during early stages of disease progression, or even under physiological conditions. Despite this, if Tau phosphorylation at MD sites is involved in neuroprotection rather than neurodegeneration, the therapeutic strategies aimed at reducing Tau and pTau could phase nothing but a mild outcome [7, 13, 14, 29, 92]. In order to appropriately design therapeutic AD strategies, the link between A*β* and Tau/pTau at the synapse needs to be fully unveiled. For example, what are the molecular mechanism leading to synapse loss? Which forms of A*β* and Tau are toxic at the synapse? Additionally, we need to better understand the exact role of soluble versus aggregated species of A*β* and Tau/pTau in physiology and pathology [100]. Alternatively, unveiling the role of Tau/pTau in modulating PCs and interneuron activity (i.e., PV+ cells) could potentially prevent cognitive decline in the latter stages of AD [101, 102]. As previously discussed, it is becoming clear that early brain network changes are announcing that the complex homeostatic network that maintains brain function has been challenged. Therefore, very early brain circuit alterations could serve as a potential biomarker for detecting the prodromal neurodegeneration stage [7, 29, 67, 68, 92]. Aiming to restore brain network function may help to prevent the latter neurodegenerative stages of AD. The rationale is not straightforward if we seek to restore brain network function during AD. Thus, understanding the basic principles of firing homeostasis in neuronal circuits is required. Perhaps a broad outlook on brain network circuit damaging processes could offer new therapeutic targets and strategies for restoring brain functions.

## Abbreviations

AD: Alzheimer's disease
APP: Amyloid precursor protein
A*β*: Amyloid-beta
pTau: Hyperphosphorylated Tau
MD: Microtubule domain
LTP: Long-term potentiation
LTD: Long-term depression
NMDAr: N-Methyl-D-aspartate receptor
AKT: Protein kinase B
GSK-3*β*: Glycogen synthase kinase-3*β*
PI3K: Phosphatidylinositol 3-kinase
CaM: Calmodulin
PP2B: Protein phosphatase 2B
PP1: Protein phosphatase 1
PSD95: Postsynaptic density protein 95
AMPK: AMP-activated kinase
PV+: Parvalbumin positive
GirK: G-protein-coupled inwardly rectifying potassium channels
RNP: Ribonucleoprotein
PC: Principal cells
NFTs: Neurofibrillary tangles.
